# Supplementary material for: Organ of Corti vibrations are dominated by longitudinal motion in vivo
Source: Commun Biol. 2022 Nov 24;5:1285. doi: 10.1038/s42003-022-04234-7 (PMC9691626; doi:10.1038/s42003-022-04234-7)
Supplement: Supplementary file 2 — reporting-summary [file 42003_2022_4234_MOESM2_ESM.pdf]

nature research

Corresponding author(s): W Dong

Last updated by author(s): 2/11/2022

Reporting Summary

Nature Research wishes to improve the reproducibility of the work that we publish. This form provides structure for consistency and transparency in reporting. For further information on Nature Research policies, see our Editorial Policies and the Editorial Policy Checklist.

Statistics

For all statistical analyses, confirm that the following items are present in the figure legend, table legend, main text, or Methods section.

n/a	Confirmed	
☐	☒	The exact sample size (*n*) for each experimental group/condition, given as a discrete number and unit of measurement
☐	☒	A statement on whether measurements were taken from distinct samples or whether the same sample was measured repeatedly
☐	☒	The statistical test(s) used AND whether they are one- or two-sided<br>*Only common tests should be described solely by name; describe more complex techniques in the Methods section.*
☒	☐	A description of all covariates tested
☒	☐	A description of any assumptions or corrections, such as tests of normality and adjustment for multiple comparisons
☐	☒	A full description of the statistical parameters including central tendency (e.g. means) or other basic estimates (e.g. regression coefficient) AND variation (e.g. standard deviation) or associated estimates of uncertainty (e.g. confidence intervals)
☐	☒	For null hypothesis testing, the test statistic (e.g. *F*, *t*, *r*) with confidence intervals, effect sizes, degrees of freedom and *P* value noted<br>*Give P values as exact values whenever suitable.*
☒	☐	For Bayesian analysis, information on the choice of priors and Markov chain Monte Carlo settings
☒	☐	For hierarchical and complex designs, identification of the appropriate level for tests and full reporting of outcomes
☒	☐	Estimates of effect sizes (e.g. Cohen's *d*, Pearson's *r*), indicating how they were calculated

Our web collection on statistics for biologists contains articles on many of the points above.

Software and code

Policy information about availability of computer code

Data collection	All data were collected using custom software developed in Matlab (R2018b) and C# (Visual Studio Dev14).
Data analysis	All data were analyzed using Matlab (R2018b)

For manuscripts utilizing custom algorithms or software that are central to the research but not yet described in published literature, software must be made available to editors and reviewers. We strongly encourage code deposition in a community repository (e.g. GitHub). See the Nature Research guidelines for submitting code & software for further information.

Data

Policy information about availability of data

All manuscripts must include a data availability statement. This statement should provide the following information, where applicable:

- Accession codes, unique identifiers, or web links for publicly available datasets
- A list of figures that have associated raw data
- A description of any restrictions on data availability

Data are available for download at figshare (https://doi.org/10.6084/m9.figshare.18217127). This statement is also included in the manuscript.

Field-specific reporting

Please select the one below that is the best fit for your research. If you are not sure, read the appropriate sections before making your selection.

☒ Life sciences ☐ Behavioural & social sciences ☐ Ecological, evolutionary & environmental sciences

For a reference copy of the document with all sections, see nature.com/documents/nr-reporting-summary-flat.pdf

Life sciences study design

All studies must disclose on these points even when the disclosure is negative.

Sample size	Sample size was determined by including all available data obtained using the relevant experimental protocols. These samples are part of a larger ongoing research project.
Data exclusions	All datasets (n=6 animals) that were obtained with relevant experimental protocols were included in the manuscript.
Replication	Individual datapoints for each dataset are shown in the manuscript. All datasets (n=6 animals) showed the same result that is discussed.
Randomization	The study includes one study group, all datasets contributed to this group.
Blinding	Investigators were not blinded. Analysis of each dataset was performed using fixed sets of parameters.

Reporting for specific materials, systems and methods

We require information from authors about some types of materials, experimental systems and methods used in many studies. Here, indicate whether each material, system or method listed is relevant to your study. If you are not sure if a list item applies to your research, read the appropriate section before selecting a response.

Materials & experimental systems

n/a	Involved in the study	
☒	☐	Antibodies
☒	☐	Eukaryotic cell lines
☒	☐	Palaeontology and archaeology
☐	☒	Animals and other organisms
☒	☐	Human research participants
☒	☐	Clinical data
☒	☐	Dual use research of concern

Methods

n/a	Involved in the study	
☒	☐	ChIP-seq
☒	☐	Flow cytometry
☒	☐	MRI-based neuroimaging

Animals and other organisms

Policy information about studies involving animals; ARRIVE guidelines recommended for reporting animal research

Laboratory animals	Meriones unguiculatus, n=6, female, age was not determined, but all animals were >3 months (=adult for this species)
Wild animals	Study did not involve wild animals
Field-collected samples	Study did not involve samples collected in the field
Ethics oversight	The care and use of animals were in accordance with guidelines of, and approved by, the Institutional Animal Care and Use Committee (IACUC) of the VA Loma Linda Healthcare System.

Note that full information on the approval of the study protocol must also be provided in the manuscript.
